# DABAM: an open-source database of X-ray mirrors metrology

**DOI:** 10.1107/S1600577516005014

**Published:** 2016-04-20

**Authors:** Manuel Sanchez del Rio, Davide Bianchi, Daniele Cocco, Mark Glass, Mourad Idir, Jim Metz, Lorenzo Raimondi, Luca Rebuffi, Ruben Reininger, Xianbo Shi, Frank Siewert, Sibylle Spielmann-Jaeggi, Peter Takacs, Muriel Tomasset, Tom Tonnessen, Amparo Vivo, Valeriy Yashchuk

**Affiliations:** a ESRF – The European Synchrotron, 71 Avenue des Martyrs, 38000 Grenoble, France; b AC2T Research GmbH, Viktro-Kaplan-Strasse 2-C, 2700 Wiener Neustadt, Austria; c SLAC National Accelerator Laboratory, 2575 Sand Hill Road, Menlo Park, CA 94025, USA; d NSLS II, Brookhaven National Laboratory, Upton, NY 11973-5000, USA; e InSync Inc., 2511C Broadbent Parkway, Albuquerque, NM 87107, USA; f Elettra-Sincrotrone Trieste SCpA, Basovizza (TS), Italy; g Advanced Photon Source, Argonne National Laboratory, Argonne, IL 60439, USA; h BESSY II, Helmholtz Zentrum Berlin, Institute for Nanometre Optics and Technology, Albert-Einstein-Strasse 15, 12489 Berlin, Germany; i Swiss Light Source at Paul Scherrer Institut, CH-5232 Villigen PSI, Switzerland; j Instrumentation Division, Brookhaven National Laboratory, Upton, NY 11973-5000, USA; k Synchrotron Soleil, France; l Advanced Light Source, Lawrence Berkeley National Laboratory, MS 15-R0317, 1 Cyclotron Road, Berkeley, CA 94720-8199, USA

**Keywords:** X-ray mirror, metrology, database, Python, statistics

## Abstract

An open-source database containing metrology data for X-ray mirrors is presented. It makes available metrology data (mirror heights and slopes profiles) that can be used with simulation tools for calculating the effects of optical surface errors in the performances of an optical instrument, such as a synchrotron beamline. A typical case is the degradation of the intensity profile at the focal position in a beamline due to mirror surface errors. This database for metrology (DABAM) aims to provide to the users of simulation tools the data of real mirrors. The data included in the database are described in this paper, with details of how the mirror parameters are stored. An accompanying software is provided to allow simple access and processing of these data, calculate the most usual statistical parameters, and also include the option of creating input files for most used simulation codes. Some optics simulations are presented and discussed to illustrate the real use of the profiles from the database.

## Abstract 2

DABAM, an open-source database of X-ray mirrors metrology to be used with ray-tracing and wave-propagation codes for simulating the effect of the surface errors on the performance of a synchrotron radiation beamline.

## 1. Introduction   

Optics simulations play an essential role in the optical design and optimization of a synchrotron radiation beamline optics. These calculations must include all the parameters that determine the final beam profile and divergences. The emittance of the source and the figure errors of the optical surfaces are among the most critical parameters (Siewert, 2013 ▸). The source characteristics are fundamental for the beamline performance: the performance of a typical beamline at a third-generation synchrotron facility in terms of microfocalization and beam coherence cannot be achieved with the same beamline at an old synchrotron. This drives the community to build lower-emittance storage rings, aiming to arrive at the ‘diffraction limit’, a regime where the natural size and divergence of the radiation originated by a ‘single electron’ dominates over the size and divergence of the electron beam (Eriksson *et al.*, 2014 ▸). In addition, requirements at free-electron lasers (FELs) have led to demands for nearly perfect optics to preserve the phase space along the full length of beamlines up to 1 km in length (Samoylova *et al.*, 2009 ▸; Yashchuk *et al.*, 2015*a* ▸). The beamline optics are actually limited by the perfection of the optical elements. Typically figure errors in focusing elements are due to fabrication and design (deviation from the ideal surfaces) and deformations due to heat load, gravity, mis-clamping of the optics in the mechanics, vibrations, *etc*. The fabrication and polishing of mirrors, gratings and multilayers has witnessed a rapid improvement in recent years. The most demanding beamlines require a finish quality at the edge of what is technologically available. In order to predict accurately the beamline performance it is necessary to describe it using an adequate model which also includes the figure errors of the optical elements. Traditionally the finish errors are divided into two groups: slope errors, characterized by 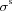, the slope error standard deviation (SD) of the slopes profile [or its root means square (RMS) of the profile with the mean slope removed], and roughness, 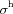, the SD value of the heights profile (or RMS of the heights profile of zero mean). For recent projects at FELs or diffraction-limited storage rings one distinguishes between the the mid- and high-spatial micro-roughness causing small- and wide-angle scatter, respectively (Harvey, 1995 ▸). Present technologies achieve values of a few angstroms or tenths of angstroms of micro-roughness and sub-microradian (down to 50 nrad) slope error. However, it is recognized that the characterization of the mirror errors by these two parameters is not complete. The SD is evaluated from the heights or slopes measurements in a collection of points on the mirror surface measured in a grid along the mirror, with points separated by a step *s* or spatial frequency 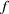 = 1/*s* (number of points per unit length). Obviously, sampling a mirror profile at different frequencies will give different values of roughness and slope errors SD. Therefore, the 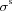 and 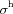 values must always be accompanied by the indication of the spatial frequency value at what they have been sampled (or the frequency interval from where they have been averaged).

For optics simulations what matters is the heights profile, described with sufficient resolution to assess the role of the different frequencies in the resulting optical image, or point spread function. Moreover, for diffraction-limited optics, the slope errors may play only a minor role (Pardini *et al.*, 2015 ▸). A frequent need in the simulation is to model the errors in the mirror profile in such a way that predicts accurately the degradation of the focal spot. Two approaches can be used. The first one is to build a profile with reasonable parameters; in other words, to ‘invent’ a mirror that would give reliable results. This is not easy because one does not know *a priori* the type of errors that will be dominant. It has been shown that too simple models like a single sinusoidal function are too naive and produce non-realistic results. Better results are achieved by randomly combining different sinusoidal signals with frequencies that are multipliers of 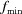 = 1/*L* (Sanchez del Rio & Marcelli, 1992 ▸). This approach has been extensively used for simulating slope errors in ray-tracing simulations of many beamlines (*e.g.* Signorato & Sanchez del Rio, 1997 ▸). The second approach for simulating a beamline is to use the mirror profile of the real mirror installed at the beamline. This is the ideal situation, but needs an existing beamline and measured mirrors, which is not the case for the typical use of optics simulations in beamline design. However, the use of an experimentally measured mirror profile is always the best solution and data from existing mirrors (or simulated or modified from the real measurements) can be used for predicting performances of beamlines under design. This approach has been used in the literature (Roling *et al.*, 2014 ▸; Siewert *et al.*, 2010 ▸; Yashchuk *et al.*, 2015*a* ▸) using data from mirrors that have been measured in the facility, or have been provided by the manufacturer. But in the general case the accessibility of good experimental data of state-of-the-art mirrors for simulations of new beamlines is difficult, because these data usually live in the computers of the metrology facilities and usually only their SD values of slopes and heights and perhaps some graphics are available, which cannot be directly used for the simulations. Simulations require the availability of the measured data, prepared with suitable preprocessing (*e.g.* detrending the main profile) and re-formatted for being read by the simulation tool.

Here, we propose to share some of the data stored in our metrology laboratories and make them available to the beamline designer. The idea is to facilitate the task of finding good data for modeling mirror errors in a realistic way to make accurate predictions for the planned optics, and compare performances of configurations with different surface errors. Although the idea of sharing metrology data in a common database is not new and has been floating in the metrology community, it was never completely implemented because of lack of manpower and coordination requiring a common and parallel effort from many laboratories. The idea of creating the open-source metrology database presented here was proposed at the 2013 Metrology, Astronomy, Diagnostics and Optics Workshop (MEADOW 2013) in Trieste. It was positively received by members of different facilities, among them the authors of this paper. They volunteered to share some data and join efforts to make these data useful for the community. The results of this action are described in this paper.

## 2. The DABAM database   

### 2.1. What is stored in the database: data and metadata   

In addition to the obvious necessity of storing the mirror profiles (heights or slopes) in the database, it is important to consider how the data, and which additional information, should be made available. The data needed for optics simulations is the mirror heights profile, usually with the main curvature removed (circular or elliptic profile) which permits to simulate and study separately the effect of surface errors and the focusing parameters (radius, ellipse semi-axes, *etc*.). It is important to note whether the metrology was a full area measurement or a single line scan measurement. At this point all the profiles stored in DABAM are single line scans. The storage of full area measurements in the database is left for a future upgrade. The instruments used for measurements are mostly the different families of slope-measuring profilers, optimized to obtain slope error data for synchrotron optics (spatial frequencies > 1 mm^−1^) such as the nanometer optical component measuring machines (NOM) (Siewert *et al.*, 2004 ▸, 2008 ▸, 2011 ▸; Alcock *et al.*, 2010 ▸; Yashchuk *et al.*, 2010 ▸; Nicolas & Martínez, 2013 ▸; Qian *et al.*, 2015 ▸), long trace profilers (LTPs) (Takacs *et al.*, 1987 ▸; Rommeveaux *et al.*, 2008 ▸; Kirschman *et al.*, 2008 ▸; Senba *et al.*, 2010 ▸) and deflectometers (Geckeler, 2006 ▸; Schulz *et al.*, 2010 ▸). Also commercially available instruments like white-light interferometers or atomic force microscopes can be used to complete surface error information for the mid- (1 mm^−1^ to 1 µm^−1^) and high- (1 µm^−1^ to 10 nm^−1^) spatial frequencies. A review of different methods for X-ray mirror metrology can be found in the paper by Takacs (2009 ▸).

It is then possible to obtain the heights profile by integration (and, *viceversa*, the slopes profile is obtained from the profile by derivation). It is necessary to detrend the main curvature and calculate the main parameters (SD values of slopes and height profiles, *etc*.). Although these calculations are not complicated from the analytical point of view, the numerical implementation and some particularities and tips vary from one laboratory to another. The final differences are not dramatic, but may be significant. A round-robin test of measurements and analysis of the data was organized among different laboratories (Assoufid *et al.*, 2005 ▸; Rommeveaux *et al.*, 2005 ▸, 2007 ▸).

Therefore, the question for the proposed database is whether we include the raw data as measured (usually slopes) or the detrended profile provided by the laboratory that provides the profile data. The raw data permit an advanced user to make the same analysis on different profiles and in this way better compare the effects. The detrended profile simplifies the use for a non-expert, who is not concerned with the technicalities of the data preparation. We decided, therefore, to store both raw and detrended data when available.

The next topic is to define which information should be available for every profile. This is the metadata. This should include a minimum set of values defining the important parameters in three families: the physical and manufacturing aspects of the mirror, the measurement and the data analysis, and results. The physical aspects include the type of mirror, its physical and optical sizes, the mirror substrate and coatings materials. In can be complemented by the year in which it was manufactured. It is very important to know which technology of surface finishing is applied in manufacturing the mirror, because each finishing technology, such as ion beam figuring (IBF), elastic emission maching (EEM), computer-aided polishing (CAP) or magneto reological finishing (MRF), will show a typical ‘fingerprint-like’ residual which is correlated to the tool-function applied to finish the mirror (Siewert *et al.*, 2012 ▸) which compare the fingerprints of CAP and EEM techniques in the power spectral density (PSD). Depending on the final finish method used, the figure error, both high frequency (roughness) and longer frequency (height error, slope error), can look very different for a given overall SD value achieved. There are two main finishing approaches: full area polishing and small area polishing. This is basically the size of the polishing tool. Full area refers to the entire polished surface to always be in contact with a polishing tool. Small area refers to IBF, EEM, MRF and CNC localized polishing techniques.

It is recommended to include the instrument used for the measurement (NOM, LTP, *etc*.). It is also interesting to include results of the treatment or detrending applied to the original measured data, and statistics from them (*e.g.* SD for heights and slopes).

It has been decided that the manufacturer of the mirror will not be mentioned in the database. The reasons for this are that we do not want to incite competition nor to bias decisions on the choice of a manufacturer based on data that by nature are incomplete. The information contained in the database is not able to answer the question of which manufacturer should be preferred. The only aim of the database is to help in the study of the effects of mirror errors in an optical system by making available experimental metrology data. The conclusions should be drawn by the ‘user’ who has the full responsibility of the result.

### 2.2. Database format, content and access   

The database is formed by a collection of mirror profiles (heights or slopes) from different facilities. The volume of the data is small, starting with a few tens of profiles, and every profile usually has a few hundred points. Also, metadata is quite light, *i.e.* only a few values and keywords. This fact, and the underlying idea to concentrate on the data and not on the container, drove us to select the simplest possible solutions for file format. We store the data in a format that is (i) human readable (in ASCII) and (ii) close to the initial raw format or what is usually supplied and exchanged by users. The use of the original raw individual files is discarded because there are multiple formats and complicate the access. We decided to use an ASCII common format. Each mirror measurement has a unique identification number, which is just the order in which it has been included in the database (*e.g.* 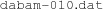). Files containing the mirror profiles have the 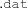 extension. A single file (database entry) can contain multiple columns which allows the raw data and the detrended data to be included. The two first columns will include the ‘default information’: the first column is always the spatial coordinate along the mirror, and the second is the main data, that can be either slope or profile, either raw or detrended. The additional information accompanying the data file is stored in an additional file with the same name and 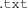 extension. This file is also ASCII and contains a list of predefined keywords and their values (*e.g.* 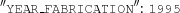). To avoid defining a new file format and to write new data parsers, we adopt a common solution for including these metadata in ASCII with the keyword-value structure: we use the *json* format (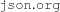).

Following these considerations, our database is a collection of files 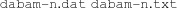, (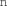 = 1,…, *N*), placed in a single place (local or remote directory) that can be accessed remotely or copied locally. Each mirror in the database is represented by two files, one containing the profile data (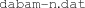) and another containing metadata (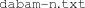), which may also include results of the analysis of the data (results of SD slopes, SD roughness, PSD, *etc*.). Initially we only concentrate on the use of one-dimensional profiles, typically from LTP or NOM measurements. However, several profiles for the same mirror can be stored using multiple columns in the 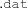 file. It is planned to extend the database to include two-dimensional or surface data with a very large number of measured points, for instance for micro-roughness characterization. As the volume of these datafiles is much larger, new solutions will be found for the storage. Appendix *A* [App appa] includes a list of the ASCII formats accepted for the data 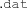 file and the description of the keywords in the metadata 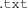 file.

### 2.3. How to contribute to the database   

The number of entries in the database is limited (25 at the time of writing this paper) but it is our intention to continue uploading new mirror profiles to the database, and to invite other metrology facilities and mirror manufacturers to submit files. The simplest way to contribute is to send ‘candidates’ mirror profiles for the database to the database maintainer. They will take care of checking that the data and metadata are the correct format before uploading them to the server. To make the submissions easier, we have implemented a web page where a user can upload the data file and define the metadata keywords in a user-friendly form (see Appendix *B* [App appb]).

## 3. DABAM in practice: extraction and processing of data from the database   

**Figure 1:**
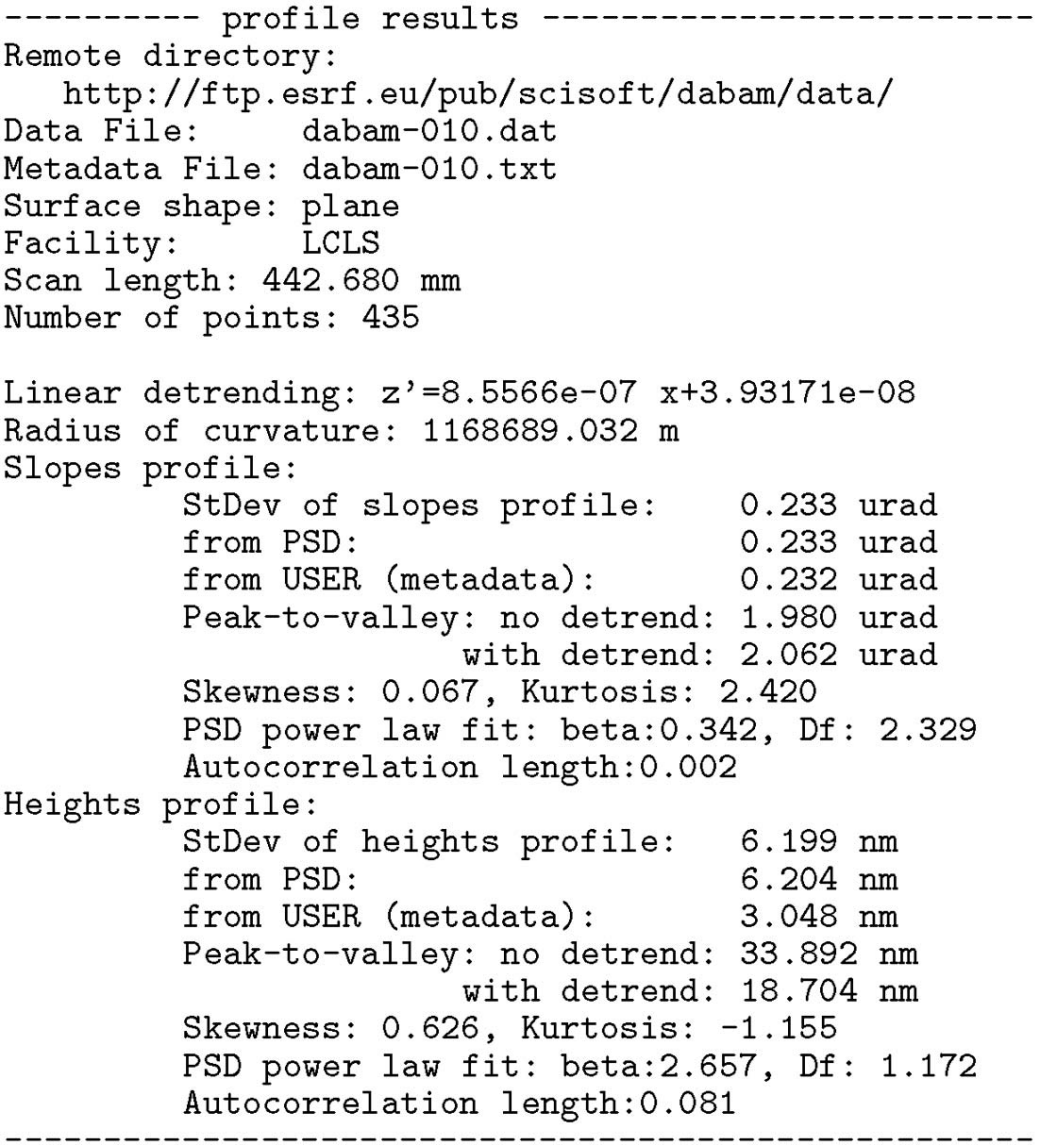
Output of the command 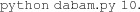

The database files described in the previous section contain all the data and information required for optical simulations. However, because the access and evaluation of the data is not straightforward, we provide a software tool that is able to access the data, perform usual operations (*e.g.* calculate SD values, plot profiles, *etc*.) and store it locally in different formats ready for simulations (*e.g.* for *SHADOW*). Again, for the sake of simplicity, we implemented this tool using the Python language, which runs on almost every machine, and is in many cases preinstalled in the machine operating system (*e.g.* Linux and MacOS). This piece of software is contained in a single file 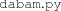 that can be dowloaded from the DABAM repositories and run by entering in a terminal window the command: 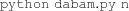, with 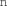 the DABAM entry number. An example is shown in Fig. 1 ▸ that shows the text information of the DABAM entry number 10. Local files are created, are easily plotted and can be exported to other programs. The behaviour of the code can be changed by using flags in the call. A description and some examples of how to call 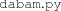 can be found in Appendix *B* [App appb].

### 3.1. Slopes and heights SD values   

In most cases the metrology apparatus records the angles α *versus* the mirror coordinate *x*. As these angles are very small, their tangent can be approximated by the angle, but the difference can be noticeable with very curved optics: 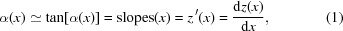
where *z* is the spatial height coordinate (thus the slope is the derivative of the height). We keep the *y* spatial coordinate along the mirror width for future use.

The slopes profile can be obtained by integration to obtain the heights profile (we use a trapezoidal rule as implemented in 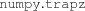),
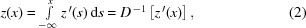
where 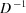 represents the anti-derivative operator. *Viceversa*, the slopes profile can be calculated by derivation of the heights profile (using Python’s 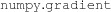).

These operations are exact in the absence of errors but have to be taken with care when using experimental profiles. The integration in the presence of random error leads to the accumulated error along the trace.

Errors also affect differentiation. The particularities of the numerical methods used also make some differences in the integration and in the Fourier transform (Yaroslavsky *et al.*, 2005 ▸).

The mean values are
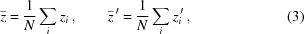
and the standard deviations (slope error SD and roughness SD, respectively):
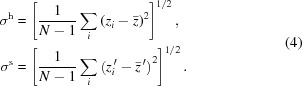



### 3.2. Main profile detrending   

In most cases the statistical values calculated from the raw measured slopes or heights profiles are meaningless because they include the main curvature of the mirror itself. In order to obtain meaningful values of slope error SD (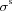) or height error SD (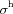) it is necessary to remove the main profile that includes the mirror curvature.

Removing the best circle radius *R* in the heights profile (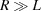) corresponds to removing a linear regression on the slopes profile. This is a very simple operation. But, again, it has to be taken with care when dealing with numerical data: the sampling of the data, the fitting method used, the error propagation, *etc*. lead to common situations where a linear fit in the slope domain and a quadratic function fit to the integrated heights profile give different radius of curvature. More generally, the slopes profile can be fitted to a polynomial of degree *d* (we use the 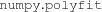 routine).

**Figure 2:**
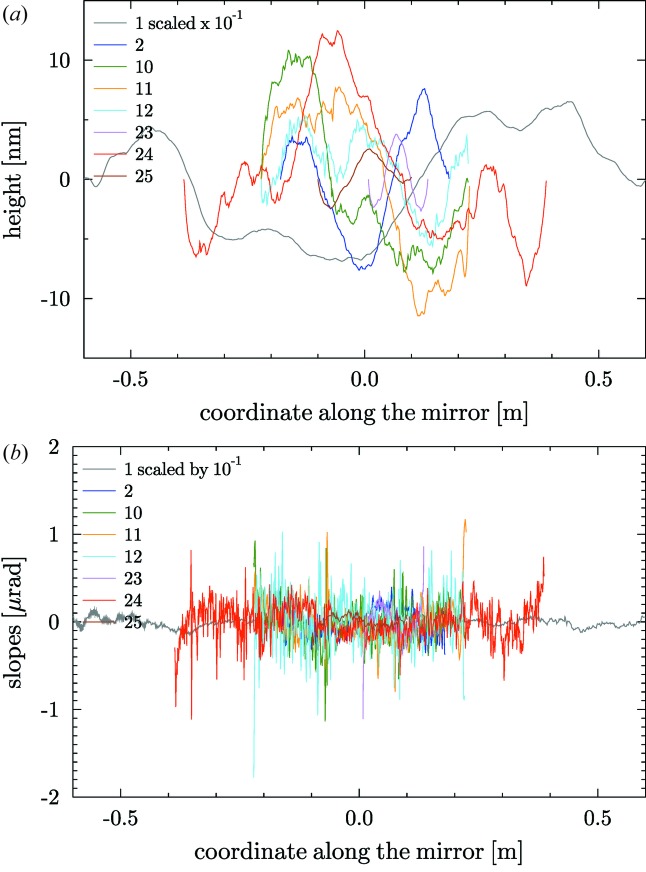
Heights (*a*) and slopes (*b*) profiles of the plane mirrors listed in Table 1 ▸.

**Figure 3:**
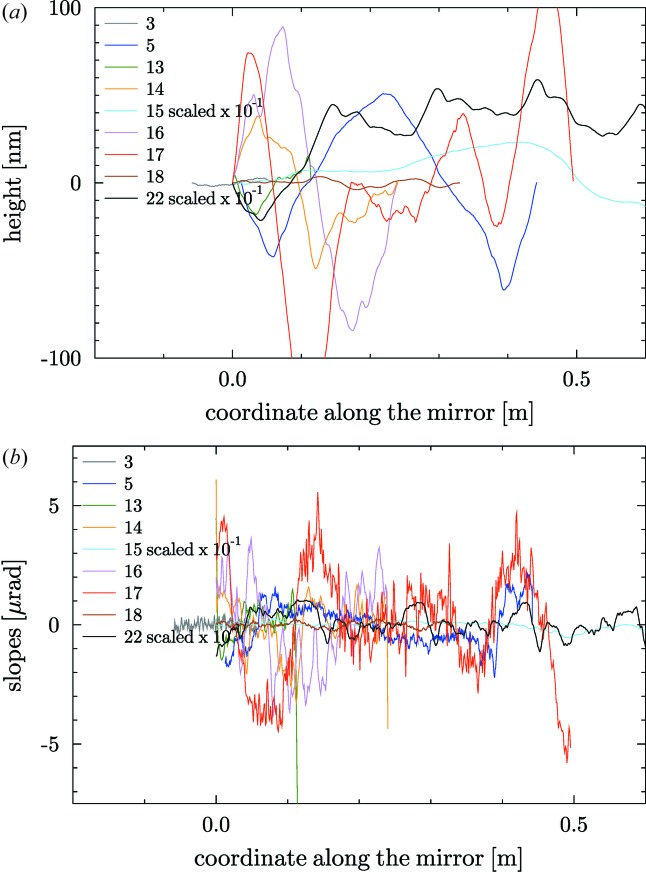
Detrended heights (*a*) and slopes (*b*) profiles of the mirrors with circular profiles (spherical, cylindrical and toroidal) listed in Table 1 ▸.

The fit is subtracted from the slopes. A linear detrending corresponds to a linear fit 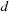 = 1 of the slopes, equivalent to fit the heights profile to a best circle with radius 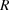 = 1/*m*, where *m* is the slope of the straight line. By default, for non-elliptical mirrors, a linear detrending of the slopes profile is systematically carried out to remove the best circle in mirrors with a circular heights profile (cylindrical, spherical or toroidal). In the case of plane mirrors, this linear detrending helps to remove a first order of a smooth approximation of other shape errors (*e.g.* the gravity sag). Fig. 2 ▸ shows the heights and slopes profiles for the plane mirrors in the database. Similarly, Fig. 3 ▸ shows the profiles of the spherical, cylindrical and toroidal mirrors (all of them with circular profile along the mirror coordinate).

**Figure 4:**
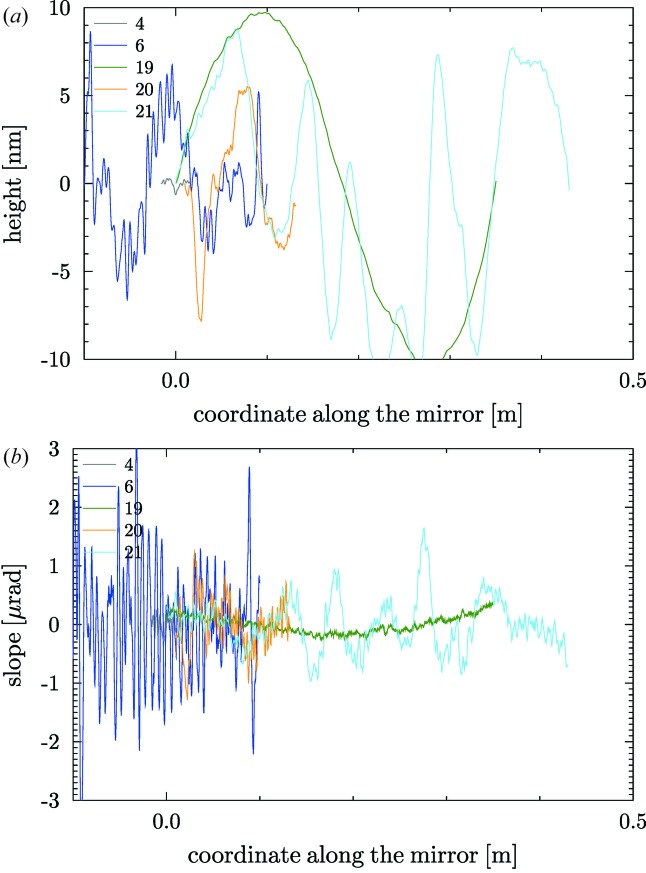
Detrended heights (*a*) and slopes (*b*) profiles of the elliptical mirrors listed in Table 1 ▸.

For elliptical mirrors, a higher degree of polynomial detrending can be applied, but it is more accurate to remove the ellipse profile wanted when the mirror was designed. This ellipse is usually described by three parameters: *p* (source–mirror center distance), *q* (mirror center–focal position distance) and θ (grazing incident angle). However, the real mirror usually follows an ellipse profile with parameters that are close but never identical to the design parameters. Therefore, an optimization of the ellipse parameters is carried out *via* a least-squares minimization to fit an optimum ellipse to the measured profile. This minimization is a local optimization method with final result depending on the initial conditions (we used the design parameters). Sometimes two more parameters are added: a constant shift in the two orthogonal directions. These parameters are highly correlated to the design parameters making the convergence to the final parameters more unstable. This fit can be performed either for the heights or the slopes profiles. As a rule of thumb, it is preferred to process data in the domain where they were recorded so one can then manipulate the residuals without producing bias to the data. Because most of our profiles are recorded in the slopes domain, and after testing different possibilities, we found it more appropriate to implement systematically in DABAM a fit in the slopes profile. Appendix *C* [App appc] describes the methodology used for the ellipse detrending. Table 1 ▸ shows the heights and slopes error values (SD) for all profiles. The heights and slopes detrended profiles are dumped in a local file with a file root defined by the user (by default 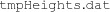 and 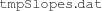). Fig. 4 ▸ shows the detrended profiles for elliptical mirrors.

### 3.3. Power spectral density analysis   

The power spectral density (PSD) for profile heights can be calculated numerically by (Church & Takacs, 1986 ▸) 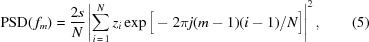
where *s* is the sampling distance in coordinates along the mirror, and 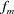 = 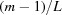 is the spatial frequency, 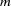 = 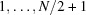, from 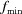 = 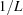 (*L* is the mirror length) to 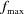 = 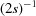, the Nyquist frequency. In a similar way, it is possible to compute the PSD of the slopes profile.

Sometimes the 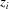 coordinates are first weighted with a window to remove border effects related to the finite profile length. Different windows have been proposed in the literature for dealing with particular filtering in different applications, but no common recommendation exists to our knowledge for the analysis of X-ray mirror data. It is important to apply windowing when the PSD is manipulated so that it removes noise. Good experience has been obtained by some of us using a Kaiser–Bessel window with different values of the strength of taper parameter. In DABAM we calculate PSDs with no window because windowing smooths the profile, thus reducing the SD of heights and slopes, and we do check these quantities after computing the PSD (see later discussion).

**Figure 5:**
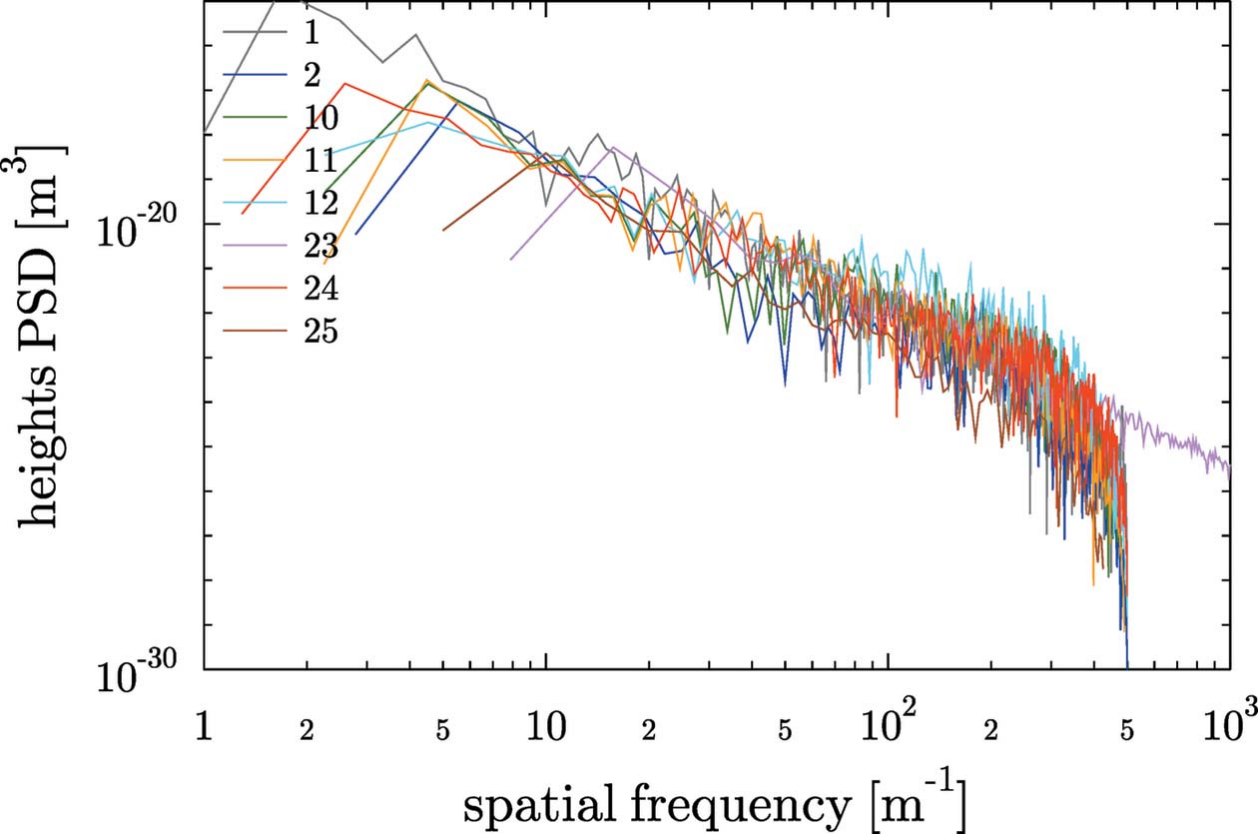
PSD of the heights profiles of plane mirrors.

The computation of equation (5) implies evaluating a sum for each value of the frequency. The result is proportional to the square of the Fourier transform modulus of the profile, and can be evaluated much faster using the fast Fourier transform algorithm implemented in most numerical libraries. With Python, equation (5) can be evaluated from a command such as 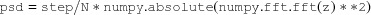
The results of heights PSD for the plane mirrors are shown in Fig. 5 ▸. As will be discussed later, all PSDs show a similar behaviour, approaching a straight line (in log–log plot) with negative slope.

As a result of the Parseval theorem, the integral of the PSD gives the second moment (variance) of the profile. Therefore, the square root of the integral of the slopes (heights) PSD gives a value equal to the standard deviation of the slopes (heights) profile (Church, 1979 ▸):
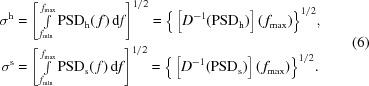
This means that the contribution of a spatial frequency *f* to the slope (height) error is proportional to the square root of the slopes (heights) power spectral density at that frequency. Roughly speaking, the average error values come from the sum of the errors for the individual frequencies (a sort of mean value). In practice, the values calculated in this way may not be exactly the same as the values computed using equation (4) because of the possible windowing and some numerical errors introduced in the calculations. A sanity test of the postprocessing operations is to check that the values calculated using equations (6) and (4) are in good agreement (Table 1 ▸).

**Figure 6:**
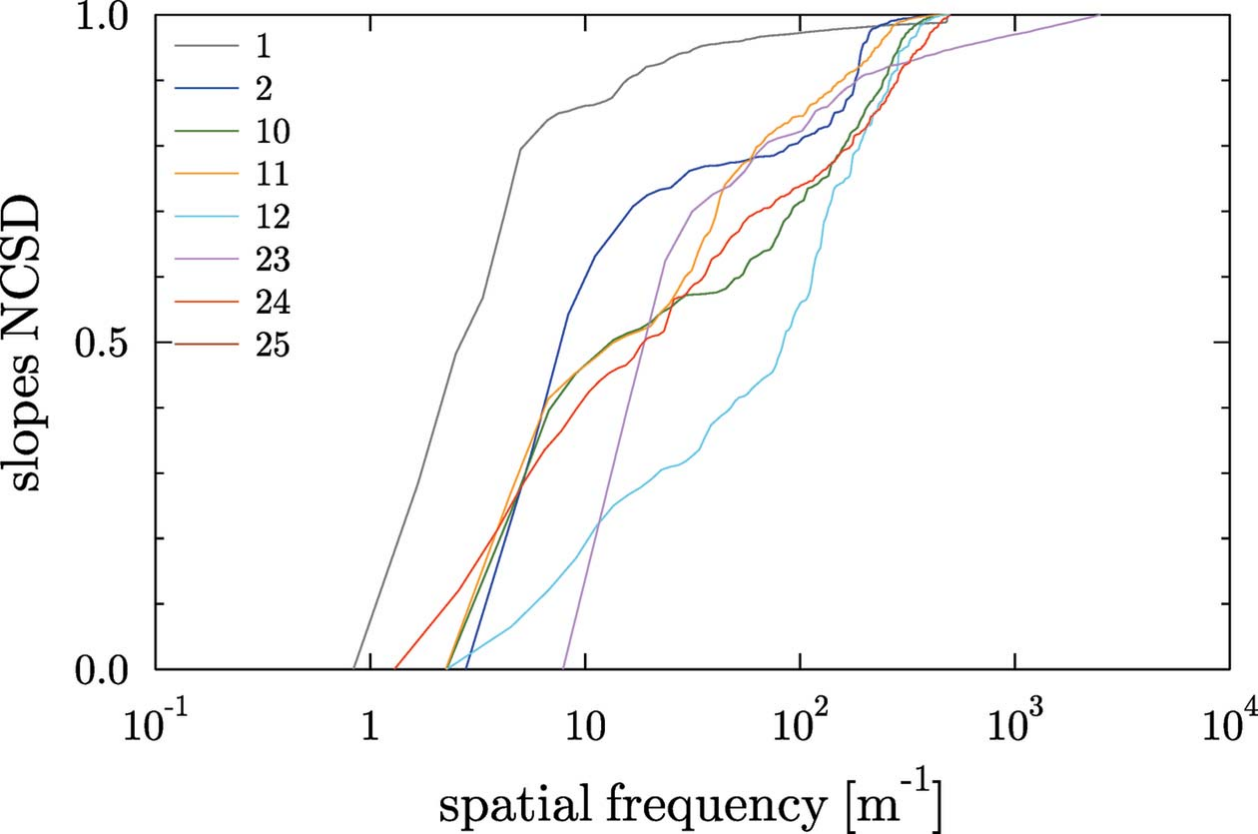
NCSD function [equation (8)] of the slopes profiles for plane mirrors.

Because of the finite nature of the acquired data and the limitation of the detector, the measured data only give a limited frequency bandwidth in the PSD. Within this bandwidth available from the measured data, the integral in equation (6) can be calculated in a limited interval of frequencies 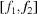 thus giving the error values due to profile irregularities in that particular range of frequencies or characteristic lengths. It is interesting to visualize the effect of how increasing frequencies contribute to the final values of profile errors. For that, it is useful to define the function CSD(*f*) (cumulative spectral density, sometimes also called cumulative spectral power):
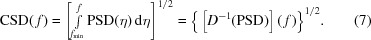
This function can be evaluated for both the heights PSD and the slopes PSD. It has a sigmoid shape, with zero value at 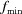 and 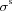 (or 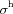) at 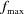. It shows the contribution to the error value of characteristic frequencies up to *f*. The point crossing 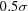 gives the frequency where higher frequencies contribute the same as lower frequencies to the final error value. It can be normalized [the normalized CSD (NCSD)],
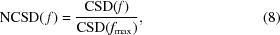
to better compare the contribution of different frequencies to the final error in different profiles. Fig. 6 ▸ shows this function for the slopes profiles of the plane mirrors in DABAM.

### 3.4. Gaussian and fractal profiles: comparison with simulated profiles   

Some statistical parameters are used to characterize heights or slopes profiles. The first moment is the mean, which is generally removed before data processing and is therefore zero. The second moment is the variance, for which the square-root is the standard deviation σ. The third moment is the skewness, which represents asymmetric spread of the height or slope distribution. The fourth moment is the kurtosis, and represents the peakedness of the distribution,
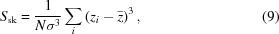


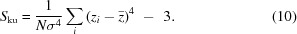



A Gaussian surface has both zero skewness and zero kurtosis. It is therefore useful to calculate these moments in order to have an idea of how our profile is separated from a Gaussian profile. The profile should be Gaussian only if the error distribution is fully random. When a deterministic polishing is used, the errors usually present a well defined pattern. Another visual way to check the Gaussianity of our profiles is to histogram the heights or slopes. The result can be fitted to a Gaussian to visually see whether the profiles adjust well or not to Gaussian profiles. Table 2 ▸ contains the skewness and kurtosis values of all detrended profiles in DABAM. The resulting values are different from zero, meaning that they are far from being normal. The mirror finish for synchrotron mirrors, as studied here, is carried out up to the nanometric level, thus revealing the atomic distribution in a superpolished surface. Therefore, the surface statistics are not expected to be Gaussian but more approaching a fractal structure.

One interesting family of profile errors have PSDs that look like a straight line in the log–log plot, which corresponds to a power law of the PSD (Voss, 1988 ▸): 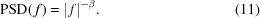
Our mirrors show a behaviour of the PSD typical of the power law. The slope of the linear fit of the log–log representation of the PSD gives 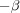. Table 2 ▸ shows these calculated values. A linear fit has been performed in the log(PSD) *versus* 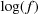 data, in a window containing over 80% of the 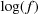 interval.

An interesting particular case of profiles presenting a power-law PSD are fractals (Church, 1988 ▸). A fractal’s shape is replicated at various length-scales. When this replication is only statistical we have a statistical fractal. Fractals are described by the so-called fractal dimension 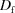, related to β by
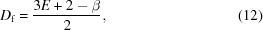
where *E* is the Euclidean dimension of our data, 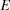 = 1 for profiles, 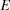 = 2 for surfaces, *etc*. For a natural fractal profile (*e.g.* coastline, *etc*.), this dimension is in the interval 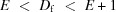, which, for one-dimensional profiles (our case), 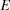 = 1.

A natural one-dimensional fractal has a fractal dimension between one and two (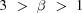), but in engineered surface and profiles it can go beyond this dimension. Looking to our profiles (Table 2 ▸), it can be observed that only a few profiles (7, 10, 12) may be true fractals because 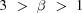. The β values we obtained are in the 3 to 5 range, meaning a lower contribution of high frequencies than expected for the natural fractal. Two possible explanations are possible.

The first explanation accounting for β values higher than for fractal profiles can be due to different types of experimental and analysis errors. The limited frequency range of the measurements implies that higher frequencies are not resolved to a good resolution and one would need a much smaller step along the mirror coordinate to better explore this zone. A lack of resolution will limit the amount of information at high frequency, therefore reducing the PSD at the high-frequency end, and increase the slope. It is remarked that the data in the log–log scale introduced non-uniformities in the distribution of the points that affect the goodness of the fit. It could be possible from a computational point of view to fit the PSDs for both heights and slopes profiles. But this is redundant; with the slopes profile being the first derivative of the heights, in Fourier space it is equivalent to multiplying by the frequency *f*, so the PSD that is the square of the Fourier transform is affected by an 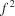 term. Thus, the power law exponent for the slopes is theoretically the exponent for heights plus two. We performed some fits using the slopes PSD and obtained values of β with some disagreement with respect to those calculated and shown in Table 2 ▸ due to errors. As a matter of fact, the error in the obtained β is large.

The other obvious explanation for accounting for large values of β and also a not very good fit to the power law is to say that our profiles are just not fractal. Church (1984 ▸) has suggested the so-called ABC model that solves some of the problems seen. A better modern approach is a method based on an autoregressive moving average (ARMA) modeling of the surface metrology (Yashchuk & Yashchuk, 2012 ▸; Yashchuk *et al.*, 2014 ▸, 2015*a* ▸,*b* ▸) resulting from a stochastic polishing process. It provides a reliable way to describe, model and parametrize the measured residual slopes profiles of the X-ray mirrors. The ARMA method is based on time-invariant linear filter (TILF) modeling of a polished surface, considered as a result of a uniform stochastic process. The ARMA/TILF model determined for the measured optics can be used to forecast a set of new surface error distributions with the same statistical properties, but with generally different parameters, such as the distribution length and slopes and heights SD. This approach has been successfully applied to numerically simulate the beamline performance of a flat offset mirror under design for using in the SASE1 beamline of the European XFEL (Samoylova *et al.*, 2009 ▸). As a future development of the DABAM tool, we plan to incorporate the corresponding codes for ARMA/TILF modeling and forecasting.

It is also interesting to calculate the autocorrelation function (ACF) of a profile 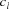. It gives the average of heights (or slopes) compared with a translated version of itself: 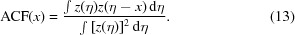
This function has a value of 1 at 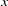 = 0 and oscillates tending to zero for large values of *x*. The autocorrelation length can be defined as the value of *x* where ACF takes the value of one half: 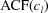 = 0.5. It gives an approximation of the length at which the profile values are uncorrelated such that they start to be random. In other words, if one expands the profile in a Fourier series, the phase of the components with spatial frequency larger than 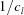 could be ‘safely’ replaced by a random phase without altering significantly the macroscopic profile shape, so keeping the same statistical properties (SD and PSD functions) and optical performances. On the contrary, if one uses a random phase for the low-frequency Fourier components (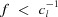), then the shape of the profile (figure errors) is significantly altered, so the optical response of the profile will be very different. The PSD and ACF provide the same information about surface statistics expressed in space frequency and space coordinates, respectively. One can pass from one to another *via* Fourier transform (Lighthill, 1958 ▸).

Statistical analysis of the real profile provides important information to create or simulate profiles with similar characteristics to the experimental ones that can be used for our simulations. Interest in playing with simulated profiles is twofold: it permits some mirror parameters to be adapted to our particular needs (*e.g.* mirror length, slope error SD, *etc.*), and allows profiles with ideal correlation (Gaussian, fractal) and SD values to be created to compare with our experimental values.

An *ab initio* simulated profile with PSD that follows the power law 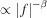 can be created using a sum of sinusoidal functions with frequencies in the desired interval and random phase, and with amplitude matching the power law: 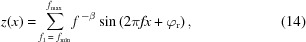
where the sum extends over a collection of *N* frequencies [usually *N* is equal to the profile points, with 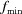 = 1/*L*, 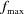 = 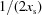, and 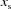 is the step along the mirror length of the mirror profile to be generated].

**Figure 7:**
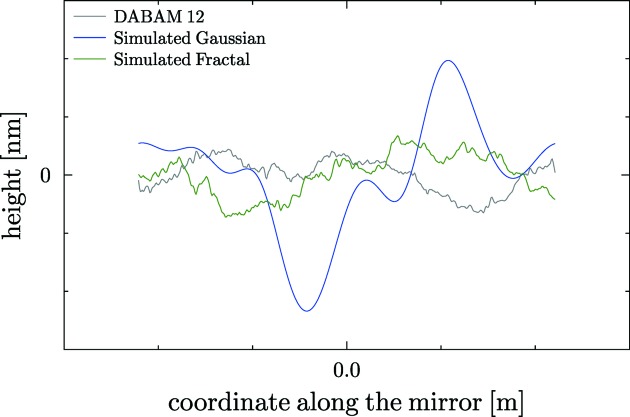
Comparison of DABAM profile 12 with a simulated fractal with the same β and a simulated Gaussian with the same 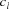.

As an example, we have used the profile entry 12 and simulated a profile with the same parameters (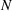 = 443, 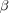 = 2.8, 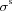 = 0.3 µrad). The original and simulated profiles and their PSD are compared in Figs. 7 ▸ and 8 ▸, respectively. From these figures, it can be appreciated that the experimental and simulated profiles have similar structure for large frequencies, but for small frequencies (figure or shape errors) are completely different, because of the random phases used. In fact, another realisation with different phases produces a profile with completely different shape errors. This illustrates the interest in having real profiles available, so justifying the DABAM database. Moreover, one can also simulate *ab initio* profiles with the Gaussian height distribution and Gaussian autocorrelation function (Garcia & Stoll, 1984 ▸). Using also the entry 12 in the DABAM data (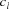 = 44 mm, 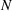 = 443, 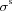 = 0.3 µrad) a simulation is performed and shown in Figs. 7 ▸ and 8 ▸. It is evident that the shape and PSD of the simulated profile are very different from the experimental one, thus concluding that our profiles are not Gaussian correlated. In the *ab initio* simulation of profiles it is important that the sampling parameters are strongly correlated to the profile inputs (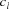 or β) and profile normalization (to 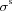 or 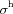). Therefore, if one wants to work using simulated profiles, it is interesting to work in tandem using the DABAM tool that permits different experimental profiles to be looked at and compared with the results of a simulation tool. Both tools are available in the *ShadowOui* package.

## 4. Examples of applications   

When calculating the performances of an optical system, two complementary methods may be used. The first is the ray-tracing approach, based on geometrical optics. The second is physical optics, that permits calculation of the intensity distribution (diffraction pattern) produced by our optics when illuminated by a coherent beam. Here we perform some simulations using DABAM profiles using simple approximations of both methods, and a full simulation combining both methods.

### 4.1. Simple ray-tracing calculations including slope errors from DABAM   

**Figure 9:**
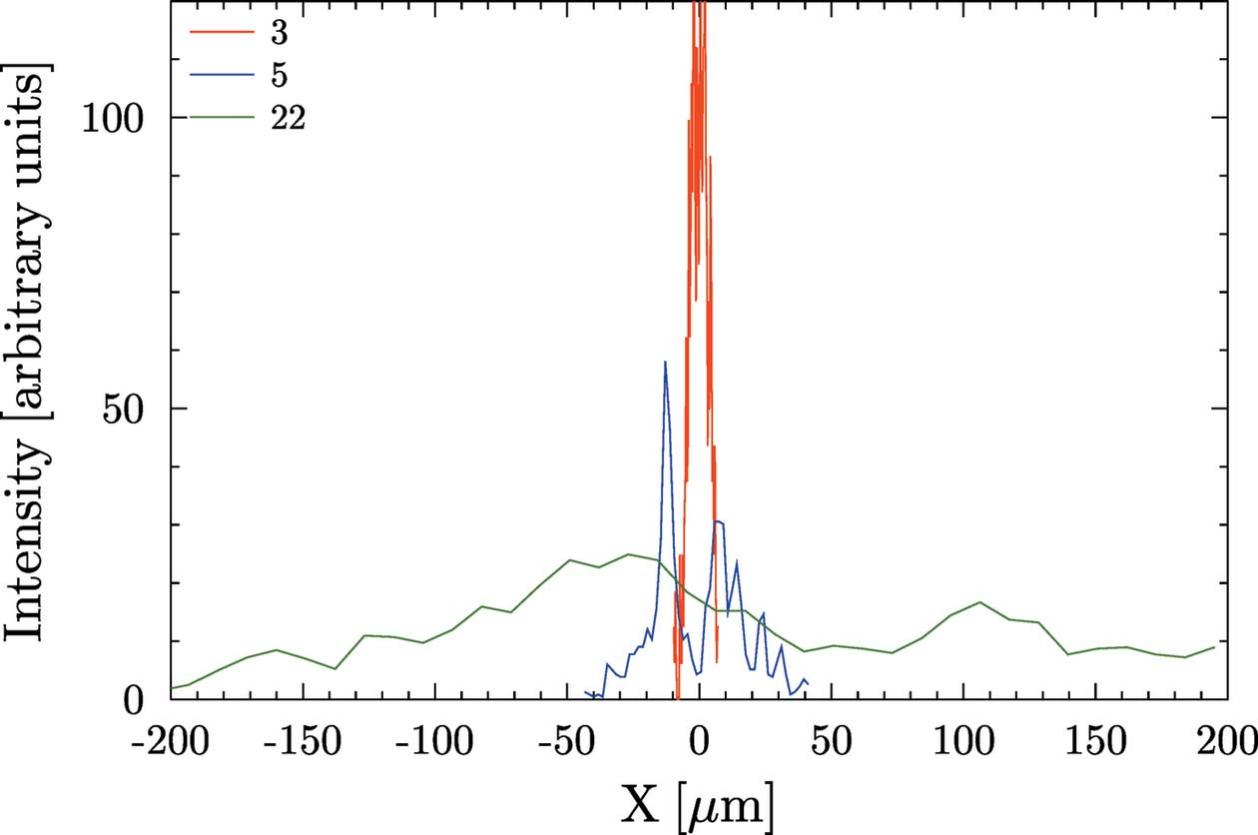
Image produced by circular mirrors set to focus with *p* = 30 m, *q* = 10 m, 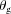 = 3 mrad with slope errors from the database entries 3 (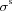 = 0.17 µrad), 5 (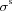 = 0.85 µrad) and 22 (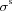 = 5.32 µrad). For clarity, the intensity of profile 22 has been multiplied by 5. The effect of higher broadening produced by profiles with high slope error (entry 22) is evident. Note also the structures in the focal intensity distribution produced by the low-frequency errors (shape or figure errors).

A simplified model for ray-tracing can be easily implemented if (i) the geometrical optics approximation is applicable, *i.e.* diffraction effects produced by the mirror aperture and mirror irregularities are negligible, (ii) one-dimensional tracing is assumed in the plane of the profile, (iii) the source can be assumed with zero dimension (point source), (iv) focusing is performed with a focalization element of focal length *F*. Let us suppose that the mirror is placed at a distance *p* from the source and the image plane is placed at *q* downstream from the mirror. The lens equation is verified for good focalization:
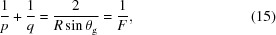
with 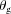 being the grazing angle on the mirror, and *R* the radius of curvature of the mirror. It is convenient to work with a coordinate *X* perpendicular to the optical axis, so the 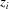 heights profile and 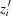 slopes profile *versus* 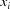 can be expressed as a function of the new coordinate 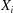 = 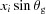, forming the following incident angles with respect to the optical axis: 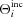 = 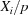. The angle after the reflection, always measured with respect to the optical axis, is obtained by adding the contribution of the mirror curvature (focusing) and slope error: 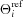 = 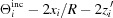, where the mirror curvature introduced a slope 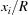 and a factor of two is set to indicate that it is measured with respect to the entrance direction. The coordinates at the image plane are just 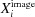 = 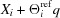, which are not uniformly distributed. The intensity profile can be obtained by a histogram of the 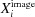 array. The intensity profiles at the image plane for some mirrors with circular shape (spherical, toroidal or cylindrical) are shown in Fig. 9 ▸.

### 4.2. Simple physical-optics calculations including slope errors from DABAM   

In a simplified model to calculate the effect of a mirror focusing including profile errors one could consider the source as a spherical wavefront (point source). The propagation of the wave in a vacuum requires solving the Fresnel or Fresnel–Kirchhoff integrals usually by Fourier optics (*e.g.* Chubar & Elleaume, 1998 ▸; Bahrdt *et al.*, 2014 ▸; Shi *et al.*, 2014*b* ▸; Kewish *et al.*, 2007*a* ▸,*b* ▸; Pardini *et al.*, 2015 ▸) or by reducing the integral to a sum over an adequate gridding of the source and image plane. The second method is used here: we sample the directions perpendicular to the optical axis at the source, mirror and image plane and calculate the propagation from one element (*e.g.* source) to the next one (*e.g.* mirror) by multiplying the electric field at the source by a phase term that depends on the optical path. Thus, we are approximating the Fresnel–Kirchhoff integral to a simple sum: 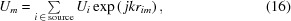
where the electric field at the source can been set to 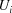 = 1, and 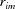 is the distance from the *i*th source position to the *m*th point position in the mirror plane.

**Figure 10:**
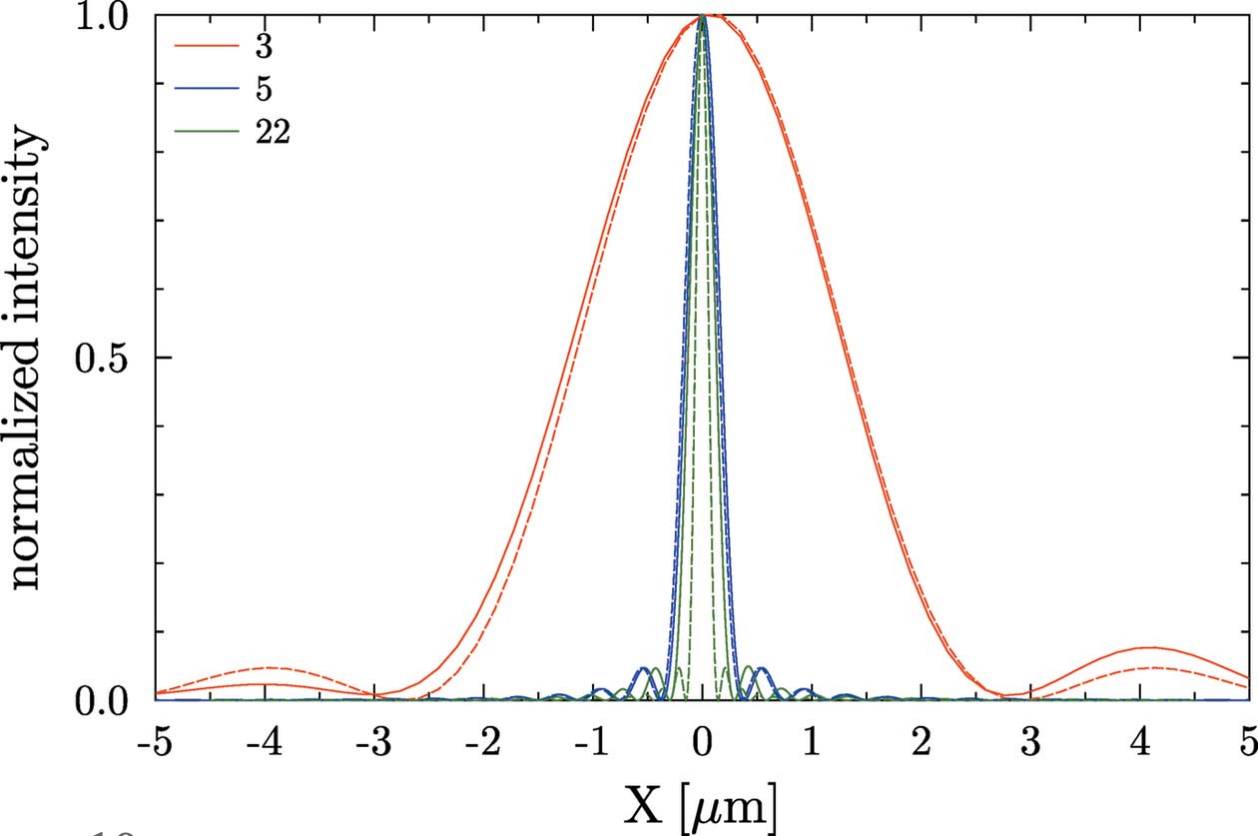
Intensity profiles produced by circular mirrors set to focus with *p* = 30 m, *q* = 10 m, θ = 3 mrad with slope errors from the database entries 3 (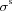 = 0.17 µrad), 5 (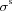 = 0.85 µrad) and 22 (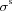 = 5.32 µrad) at a photon wavelength 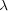 = 1 Å. The dashed line corresponds to the system without errors, and the solid line used the experimental profile after detrending. The effect of broadening of entry 3 with respect to the others is evident because of its smaller aperture 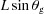. The Airy disk values [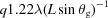] are: 3.45, 0.46 and 0.18 µm for profiles 3, 5 and 22, respectively. Note also that the widths of the intensity profiles for entries 5 and 22 are much smaller than the widths calculated by ray tracing (Fig. 9 ▸), indicating that in these cases the effect of beam coherence and diffraction are negligible. This is no longer true for entry 3, where both geometrical optics and physical optics contributions have comparable widths.

The focusing effect of the mirror is modeled as a thin element that changes locally the phase by 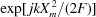, where *F* is given in equation (15) and 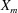 is the distance to the optical axis, as defined in the previous section, and *k* = 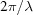. The effect of the slope errors is modeled by an additional phase term 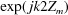, where 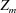 = 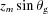 is the heights profile projected onto the plane perpendicular to the optical axis (and interpolated onto the grid in use). Therefore, the combined effect of mirror focusing and slope errors changes the electric field amplitude,
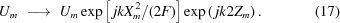
After calculating the electric fields 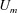 at the mirror plane including the mirror effects (focusing and profile errors) a propagation from mirror to image plane is performed by applying again equation (16). The intensity is then evaluated at the image plane as the square modulus of the electric field amplitude. Using this method it is possible to obtain a good approximation of the effect of the profile errors in a focusing mirror. Results of some simulations are shown in Fig. 10 ▸.

### 4.3. Hybrid (ray-tracing and physical-optics) calculations   

**Figure 11:**
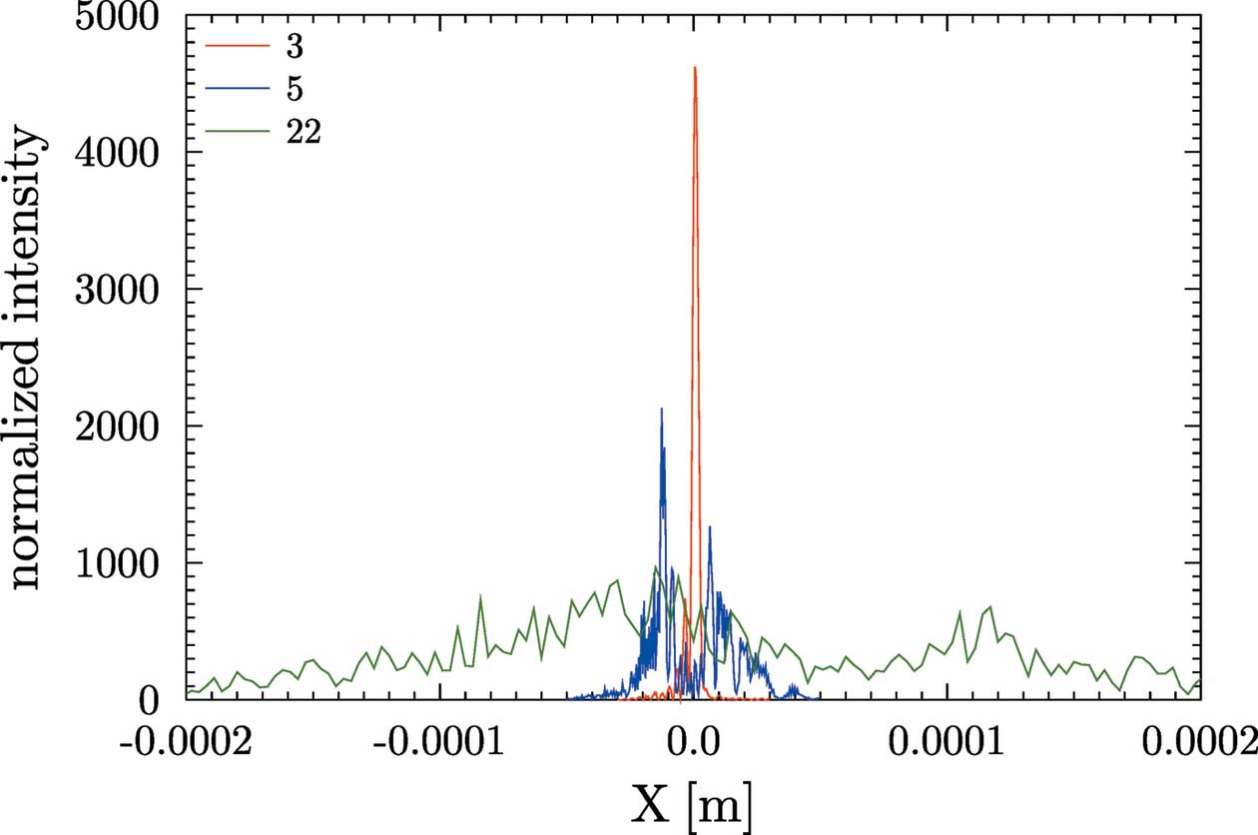
Intensity profiles produced by circular mirrors set to focus with *p* = 30 m, *q* = 10 m, θ = 3 mrad with slope errors from the database entries 3 (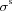 = 0.17 µrad), 5 (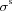 = 0.85 µrad) and 22 (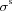 = 5.32 µrad) at a photon wavelength 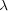 = 1 Å. Simulations are performed using hybrid ray tracing and physical optics algorithms (Shi *et al.*, 2014*b* ▸).

For many cases in new X-ray sources with particular regard to diffraction-limited storage rings and free-electron lasers, ray-tracing is insufficient for giving a good estimate of the distribution of the intensity at the focal position (Pardini *et al.*, 2015 ▸), since it does not include coherent effects due to the mirror aperture and the mirror imperfections. On the other hand, wave-optics simulation using single wavefront propagation (plane, spherical waves or single electron emission) is usually not accurate because the synchrotron beam is not fully coherent and, moreover, is not diffraction-limited [the product of the source size *s* and source divergence 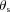 does not verify 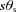 ≃ 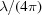, with λ the photon wavelength]. For these cases, an intelligent combination of geometric and physical optics gives reasonable results. This method has been proposed (Shi *et al.*, 2014*a* ▸) and implemented in *SHADOW* (Shi *et al.*, 2014*c* ▸). Results of simulations for the mentioned case with the *SHADOW* hybrid method are given in Fig. 11 ▸. For cases in the geometrical optics regime (*e.g.* entries 5 and 22), the intensity profiles are similar to that anticipated using the simplistic ray-tracing model. In the case of entry 3, the hybrid calculation provides more accurate results since it includes all effects of geometry and broadening of the focal image due to diffraction and interference.

### 4.4. DABAM integration in simulation packages: *ShadowOui*   

**Figure 12:**
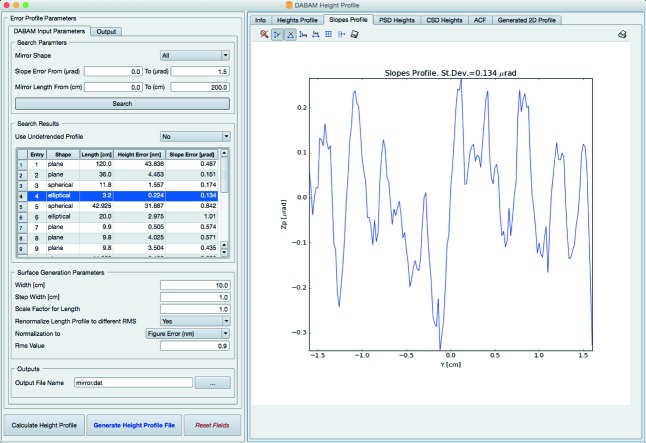
Interface of DABAM as available in *ShadowOui*.

The availability of DABAM and its Python binding makes it suitable for integration into simulation environments that can interact with Python. Oasys is a Python-based environment (Sanchez del Rio *et al.*, 2014 ▸) designed to integrate optics simulations into virtual experiments. *ShadowOui* is the Oasys user interface for the ray-tracing *SHADOW*, also including the hybrid approach used before. The DABAM database is fully integrated into *ShadowOui* and the user can automatically access and use the metrology files for simulations in an automatic and transparent way. Fig. 12 ▸ shows the *ShadowOui* widget dedicated to access DABAM and insert the slopes errors into the *SHADOW* optical elements. *ShadowOui* also includes a profile simulator that is able to create synthetic surfaces by using experimental or synthetic (fractal or Gaussian) profiles and then combine them into a two-dimensional surface by defining the geometry of a transversal profile.

## 5. Summary and conclusions   

A collaborative effort among different metrology laboratories of several institutions has permitted collection of a set of measured mirror profiles that are grouped and stored in a database. This information is open to the community to allow the role of mirror irregularities and errors in the performances of optical systems to be assessed, mostly in synchrotron beamlines. In addition to the experimental data, software is written to retrieve and process the mirror profiles in the database. Some statistical parameters are calculated on the detrended profiles. Moreover, using simple methods of propagation based on geometrical optics (ray-tracing) and physical optics, we can compute the effect of the profile errors in a simple focusing configutarion.

The DABAM database is not centralized, and local copies or other servers can be deployed. In particular, the DABAM concept can also be used to store all data measured in a metrology laboratory but keeping the server and access private.

The main use of the DABAM data is in combination with simulation tools, like ray tracing. The *ShadowOui* package incorporated in the hybrid model contains tools to access DABAM data and to simulate *ab initio* surfaces. The DABAM database can easily be integrated into other simulation tools.

## Figures and Tables

**Figure 8:**
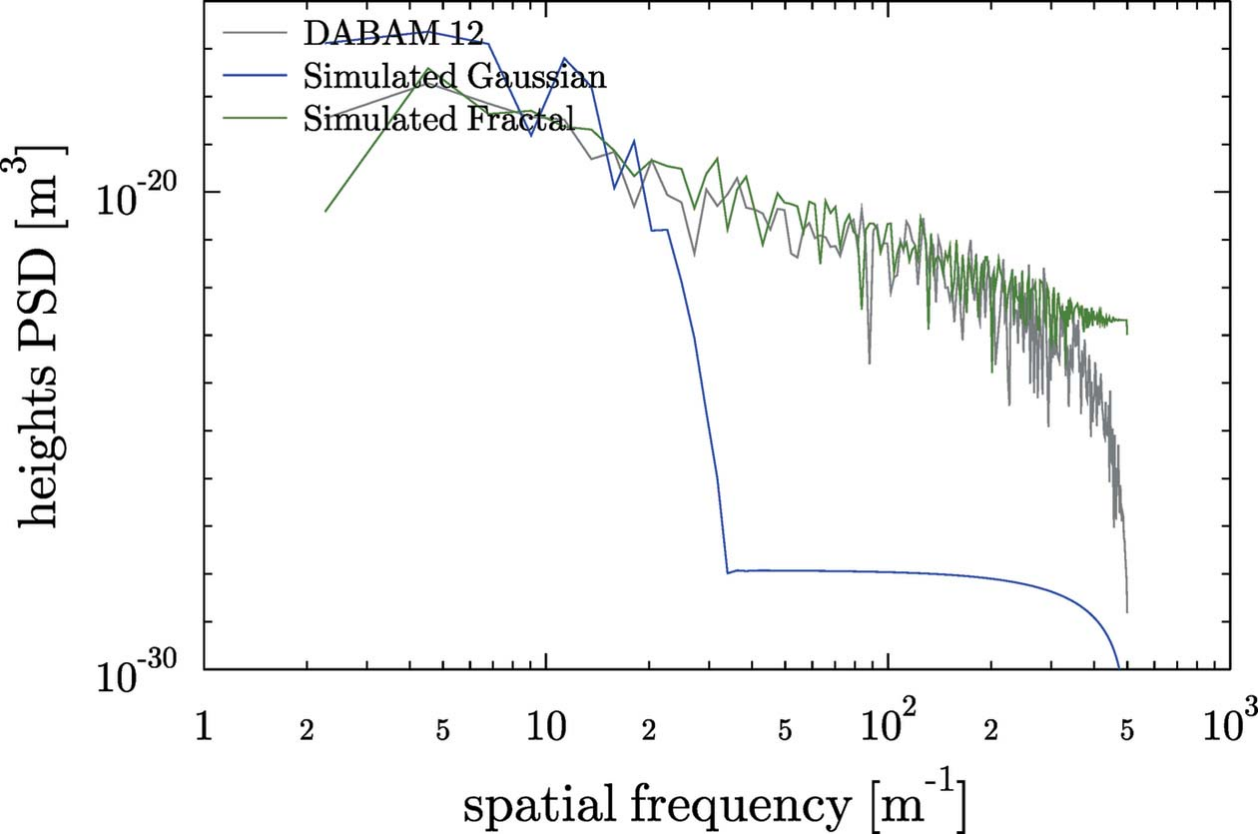
Comparison of the PSD of DABAM profile 12 with a simulated fractal with the same β and a simulated Gaussian with the same 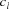.

**Figure 13:**
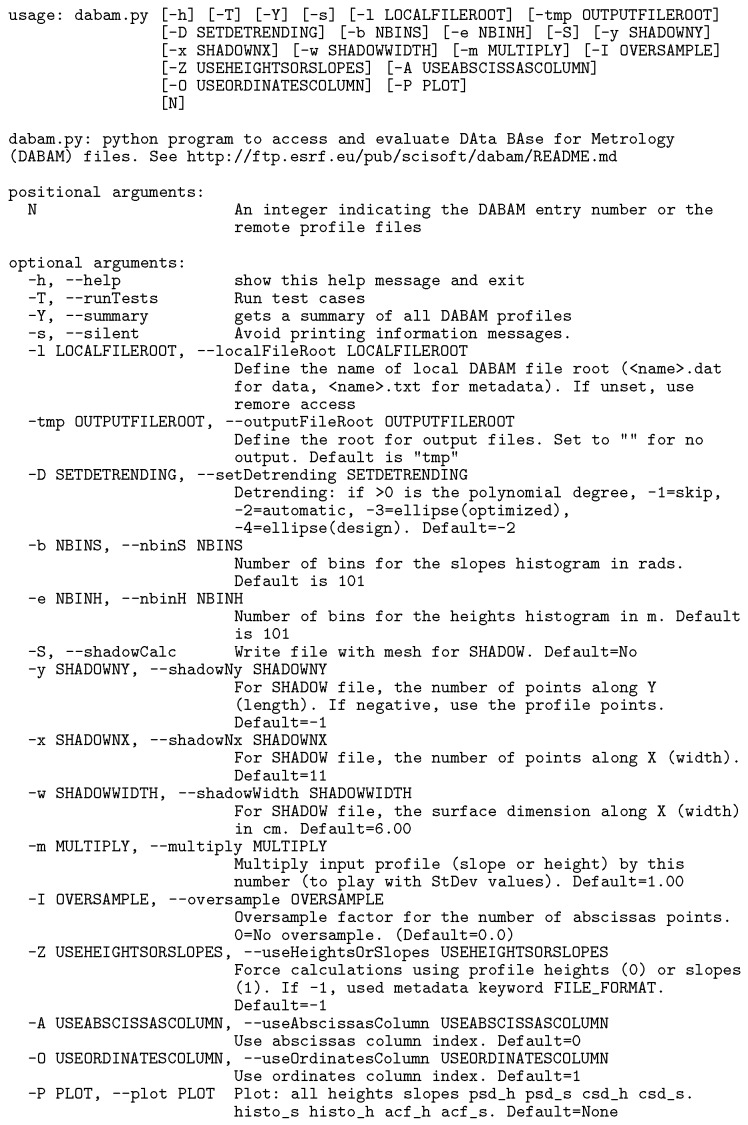
DABAM help: output of the command 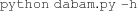.

**Table 1:** Calculated values of slope error 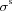 (in µrad) and height error 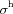 (in nm) for the different database data calculated from the points of the heights or slopes profiles, respectively The first value in parentheses is calculated by integrating the PSD. The second value in parentheses, where available, is the value provided by the user that supplied the data. Scan length (*L* in mm) and mirror shape are also included.

Index	Shape	*L*	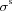	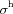
1	Plane	1200	0.49 (0.49)	43.85 (43.85)
2	Plane	360	0.15 (0.15)	4.46 (4.46)
3	Spherical	118	0.17 (0.17)	1.56 (1.56)
4	Elliptical	32	0.13 (0.13)	0.22 (0.22)
5	Spherical	429	0.84 (0.84)	31.89 (31.90)
6	Elliptical	200	1.01 (1.01)	2.98 (2.98)
7	Plane	99	0.58 (0.57)	0.57 (0.51)
8	Plane	97	0.57 (0.57, 0.67)	4.05 (4.05, 0.28)
9	Plane	97	0.44 (0.44, 0.61)	3.53 (3.52, 0.41)
10	Plane	442	0.23 (0.23, 0.23)	6.20 (6.20, 3.05)
11	Plane	445	0.23 (0.23, 0.23)	6.58 (6.59, 3.23)
12	Plane	442	0.32 (0.32, 0.32)	2.93 (2.86, 2.51)
13	Spherical	114	1.30 (1.29, 1.51)	9.11 (8.97, 10.86)
14	Spherical	240	1.15 (1.14, 1.05)	23.38 (23.28, 15.37)
15	Toroidal	800	2.13 (2.13, 2.13)	170.87 (170.87, 97.36)
16	Toroidal	239	1.85 (1.84, 1.86)	54.67 (54.78, 36.54)
17	Toroidal	495	2.27 (2.27, 2.40)	61.49 (61.54, 47.58)
18	Cylindrical	330	0.12 (0.12, 0.12)	1.83 (1.83)
19	Elliptical	350	0.14 (0.14, 0.06)	7.25 (7.25)
20	Elliptical	121	0.45 (0.45, 0.50)	3.40 (3.39)
21	Elliptical	430	0.43 (0.43, 0.50)	6.05 (6.05)
22	Cylindrical	1130	5.32 (5.32, 5.00)	400.83 (400.82)
23	Plane	127	0.18 (0.18, 0.18)	2.17 (2.17)
24	Plane	774	0.20 (0.20, 0.20)	5.35 (5.35)
25	Plane	200	0.07 (0.07, 0.07)	1.48 (1.47, 1.33)

**Table 2:** Calculated values of skewness *S*_sk_, kurtosis *S*_ku_, correlation length *c*_l_ (in mm) and β [equation (11)] for the first 25 DABAM profiles *N* is the number of points of the profile.

		Heights				Slopes	
Index	*N*	*S*_sk_	*S*_ku_	*c*_l_	β	*S*_sk_	*S*_ku_
1	1201	−0.17	−1.45	161	3.89	−0.25	0.06
2	361	−0.25	−1.12	51	3.24	−0.02	−0.46
3	591	0.42	−1.35	24	4.79	−0.16	−0.37
4	161	−1.16	0.74	3	4.61	−0.19	−0.52
5	1718	0.01	−1.15	61	4.38	0.17	−0.54
6	801	0.34	−0.35	14	4.87	−0.11	1.10
7	100	−0.02	−0.02	3	0.97	0.02	0.68
8	99	0.71	−0.30	10	5.35	−0.21	−0.71
9	99	0.84	−0.69	17	5.14	1.06	1.89
10	435	0.63	−1.16	81	2.66	0.07	2.42
11	438	−0.48	−1.48	91	3.26	1.50	6.74
12	443	−0.59	−0.64	43	2.76	−0.42	2.37
13	115	−0.18	−1.01	19	3.03	−3.01	19.08
14	241	0.01	−1.05	37	3.70	−0.22	3.39
15	801	−0.59	−0.67	126	4.34	0.22	0.68
16	240	0.02	−1.37	48	4.55	−0.12	−1.02
17	496	−0.57	0.39	38	3.99	−0.24	−0.47
18	661	−0.29	−0.62	22	3.86	−0.27	−0.42
19	1751	−0.08	−1.55	73	3.23	0.36	−0.71
20	244	0.06	−0.59	14	3.51	−0.02	0.23
21	2151	−0.37	−1.15	22	3.94	0.58	0.98
22	2261	−0.31	−1.40	211	4.30	−0.13	−0.30
23	636	0.38	−1.42	18	3.60	−0.37	3.04
24	775	0.78	−0.30	78	3.09	−0.37	2.25
25	171	−0.12	−1.02	32	4.02	−1.21	3.33

## Appendix A Format of data files and metadata keywords

The data files stored in the database contain one-dimensional profiles of measured mirrors. They are presented as ASCII files named  with some header lines and a block of numeric data arranged in multiple columns. Usually, the data are the undetrended raw profiles, but detrended profiles can be used instead or in addition (using new columns). The file format accepted by the  code are numbered and are:

Spatial coordinates in first column (column index 0), measured slope in next column (column index 1). Additional columns contain other information (*e.g.* detrended profile) using the same abscissas column, thus the column structure is .

. The same as  but using measured heights in column index 1.

. Multi-abscissas and multicolumn slope profiles, arranged like . This format is interesting for including several measured profiles in the same DABAM entry, each of them may have a different abscissas gridding.

. The same as  but using measured heights in *Y* columns.

Metadata (additional information on the mirror) are arranged in a separated file named  that contains a collection of keywords stored in ASCII  format. Keywords that must be filled for all entries (mandatory) are shown in . A ‘null’ assignment means an undefined keyword. New keywords can be added by the user but will be ignored by :

. Type of file used.

. Number of header lines in the data file.

. Conversion factor from user units to SI units for the column index 0.

. Conversion factor from user units to SI units.

. Year of fabrication of mirror.

. Type of mirror (toroidal, sagittal cylinder, elliptical, *etc*.).

. Mirror function (white beam mirror, collimating mirror, focusing mirror).

. Physical size in SI units (m).

. Optical size in SI units (m) (usually the scan length).

. Mirror substrate.

. Mirror coating.

. Instrument type used for measuring data (full area or single scan like LTP, NOM, *etc*.).

. Facility, laboratory or company where the data have been measured.

. Polishing/finish technology (IBF, EEM, CAP, MRF, *etc*.).

. Environment (clamped, gravity direction, cooling system, *etc*.).

. Date of the measurement in format YYYYMMDD.

. Information for default plots (not implemented).

. Heights RMS value obtained by the user in SI units.

. Slopes RMS value obtained by the user in SI units.

. A text description or reference.

. Name and email of the user that added the profile.

. Example of other keywords that can be added by users.

## Appendix B DABAM web addresses and basic use

All entry points for DABAM use (accessing, downloading, profile submission) can be obtained from http://ftp.esrf.eu/pub/scisoft/dabam/readme.html. The DABAM files can be accessed remotely (viewed, downloaded, *etc*.) from the official address: http://ftp.esrf.eu/pub/scisoft/dabam/. In order to run the code to access and process DABAM files, the Python code  should be downloaded from: http://ftp.esrf.eu/pub/scisoft/dabam/code/dabam.py. From a terminal or console window in the local system, copy or move  to the current directory and enter  for a list and description of the options (see output in Fig. 13 ▸).

The  program accesses the data entry from the FTP repository, but access to local files is possible (use flag -l). By default, the program reads mirror spatial coordinates from the first column (column index 0) (use -A to change). The default ordinates are slopes (if metadata keyword FILE_FORMAT:1) or heights (if metadata keyword FILE_FORMAT:2), that can be overwrite by using flags -A (abscissas). The ordinates are read from the next column (column index 1) unless specified by -O (ordinates). Input coordinates are transformed to SI by applying a multiplicative factor (X1_FACTOR, Y1_FACTOR, *etc*.). Detrending is applied by default (-D -2) consisting of linear detrending for plane and circular (spherical, toroidal and cylindrical) mirrors, or elliptical detrending if the mirror is elliptic. No detrending is done if set -D -1.

The web address for profile submission is also listed in http://ftp.esrf.eu/pub/scisoft/dabam/readme.html, as well as other resources for advanced users and developers.

## Appendix C Elliptical detrending

An elliptical mirror (Howells *et al.*, 2000 ▸) is usually designed by giving the incident grazing angle of the incident beam  and the focal positions: source–mirror distance *p* and mirror–focus distance *q*. The semi-axes *a* and *b* of the ellipse are:

where 2*F* is the distance from source to focus, and ∊ is the ellipse eccentricity. In the reference frame  where the ellipse is centered, the equation of the ellipse is:

The mirror center is at

The local axes related to the mirror  are defined by a vector normal to the ellipse at ,

the normalized vector and its perpendicular (tangent to the ellipse, thus zero slope at the mirror center) are

The ellipse in the local reference system  can be written as a conic:

where

This gives a second-degree equation in *y* with coefficients

One can obtain the height *y* for a particular *x* by solving the second-degree equation  =  with  =  and the slope is  = . This permits to compute numerically the heights and slopes profiles of an ellipse defined by  in the local frame of the mirror. Usually these parameters are sligntly changed to better fit the experimental profile by a numerical local optimization. This is done in  using the  function of .
